# *MdbHLH130*, an Apple bHLH Transcription Factor, Confers Water Stress Resistance by Regulating Stomatal Closure and ROS Homeostasis in Transgenic Tobacco

**DOI:** 10.3389/fpls.2020.543696

**Published:** 2020-10-09

**Authors:** Qiang Zhao, Zihao Fan, Lina Qiu, Qinqin Che, Ting Wang, Yuanyuan Li, Yongzhang Wang

**Affiliations:** ^1^Shandong Collaborative Innovation Center of Fruit & Vegetable Quality and Efficient Production, College of Horticulture, Qingdao Agricultural University, Qingdao, China; ^2^Editorial Office of YanTai Fruits, Yantai Academy of Agricultural Sciences, Yantai, China; ^3^College of Horticulture Science and Engineering, Shandong Agricultural University, Tai-An, China

**Keywords:** apple, bHLH transcription factor, *MdbHLH130*, water stress, stomatal closure, ROS-scavenging

## Abstract

Drought is a major environmental factor that significantly limits crop yield and quality worldwide. Basic helix-loop-helix (bHLH) transcription factors have been reported to participate in the regulation of various abiotic stresses. In this study, a bHLH transcription factor in apple, *MdbHLH130*, which contains a highly conserved bHLH domain, was isolated and characterized. qRT-PCR and *P_MdbHLH130_::GUS* analyses showed that *MdbHLH130* was notably induced in response to dehydration stress. Compared with the wild-type (WT), transgenic apple calli overexpressing *MdbHLH130* displayed greater resistance to PEG6000 treatment. In contrast, the *MdbHLH130-Anti* lines were more sensitive to PEG6000 treatment than WT. Moreover, ectopic expression of *MdbHLH130* in tobacco improved tolerance to water deficit stress, and plants exhibited higher germination rates and survival rates, longer roots, and lower ABA-induced stomatal closure and leaf water loss than the WT control. Furthermore, overexpression of *MdbHLH130* in tobacco also led to lower electrolyte leakage, malondialdehyde contents, and reactive oxygen species (ROS) accumulation and upregulation of the expression of some ROS-scavenging and stress-responsive genes under water deficit stress. In addition, *MdbHLH130* transgenic tobacco plants exhibited improved tolerance to oxidative stress compared with WT. In conclusion, these results indicate that *MdbHLH130* acts as a positive regulator of water stress responses through modulating stomatal closure and ROS-scavenging in tobacco.

## Introduction

Plants are inevitably challenged by environmental factors during their life cycle (including biotic and abiotic stresses), and drought is a major stressor because it limits plant growth and crop quality and productivity (Umezawa et al., 2006; Singh and Laxmi, 2015). To survive, plants must evolve a wide range of physiological and biochemical processes for reducing water loss or increasing tolerance under drought stress (Shinozaki et al., 2003; Basu et al., 2016; Rasheed et al., 2016).

Previous studies in *Arabidopsis*, rice, and other plants have shown that the transcript levels of many drought-inducible genes are significantly increased or decreased in response to drought stress (Shinozaki and Yamaguchi-Shinozaki, 2007; Buscaill and Rivas, 2014). In *Arabidopsis*, drought-inducible genes can be classified into two groups. The first group consists of functional proteins, including water channel proteins, detoxification enzymes, key osmolyte biosynthesis enzymes, and proteases. The second group consists of regulatory proteins, including transcription factors (TFs), protein kinases, and protein phosphatases (Liu et al., 2014; Joshi et al., 2016). Among the various regulatory genes, TFs act as key regulators to transduce stress signals and regulate stress-associated target gene expression to protect plants from drought stress damage (Singh and Laxmi, 2015). In plants, many TFs, including APETALA2/ethylene-responsive element binding protein (AP2/EREBP), basic leucine zipper (bZIP), NAM/ATAF1, 2/CUC2 (NAC), nuclear factor-Y (NF-Y), basic helix-loop-helix (bHLH), dehydration-responsive element-binding (DREBs), ABA-responsive element-binding (AREBs), myeloblastosis (MYB), and WRKY, were identified in response to drought stress *via* microarray analyses (Tran et al., 2004; Sakuma et al., 2006; Nakashima et al., 2009; Ishida et al., 2012; Nuruzzaman et al., 2013; Osakabe et al., 2014; Kudo et al., 2017). The bHLH protein family, one of the largest families of TFs, is present in all eukaryotic organisms. The family is defined by the bHLH domain, which contains 50–60 amino acids and two functionally distinct regions: the 13–17 basic region located at the N-terminus for binding to specific E-box (CANNTG) motifs, and the HLH region, which forms two amphipathic *α*-helices separated by a loop of variable length for forming homodimers or heterodimers with its partners (Toledo-Ortiz et al., 2003; Feller et al., 2011).

bHLH proteins are one of the important families of conserved TFs that regulate many cellular processes in both plants and animals, and they are also involved in responses to biotic and abiotic stresses (Toledo-Ortiz et al., 2003; Castilhos et al., 2014; Sun et al., 2018; Zhao et al., 2018). In animals, bHLHs are involved in many physiological and developmental processes, such as responses to environmental signals, neurogenesis, myogenesis, sex differentiation, and cell differentiation (Atchley and Fitch, 1997; Tomita et al., 2000; Nieto et al., 2001; Stevens et al., 2008). In plants, bHLH TFs play key roles in the regulation of seed germination (Penfield et al., 2005); flowering time (Kumar et al., 2012); hypocotyl growth (Lorrain et al., 2008); trichome initiation (Qi et al., 2011); anthocyanin biosynthesis, ﬂavonoid synthesis (Ramsay and Glover, 2005); light signaling (Leivar et al., 2008); hormone signaling (Abe et al., 2003; Fernández-Calvo et al., 2011); responses to wounding, drought, salt, and low temperatures (Sun et al., 2018); iron deficiency, and phosphate starvation (Ling et al., 2002; Zhao et al., 2016). In *Arabidopsis*, there are 162 genes encoding bHLHs, and 80% have not yet been functionally characterized (Pires and Dolan, 2010; Feller et al., 2011). Recently, mounting evidence has shown that bHLH TFs are important for plant responses to abiotic stresses. For example, *AtICE1* and *MdCIbHLH1* increase cold stress tolerance in *Arabidopsis* and apple (Feng et al., 2012); *bHLH122* regulates resistance to drought, salt, and osmotic stress, and increases cellular ABA levels in *Arabidopsis* (Liu et al., 2014); *bHLH104* and *MdbHLH104* increase iron deficiency tolerance in *Arabidopsis* and apple (Zhang et al., 2015; Zhao et al., 2016); *AtMYC2* increases drought resistance by positively regulating the transcription of the drought-responsive gene *RD22* in *Arabidopsis* (Abe et al., 2003); *AtbHLH112* is a transcriptional activator that mediates proline biosynthesis and reactive oxygen species (ROS)-scavenging pathways to enhance abiotic stresses tolerance; *OsbHLH148*, an *AtMYC2* homolog, improves drought tolerance in rice (Seo et al., 2011); *NtbHLH123* increases cold tolerance by regulating the *NtCBF* genes and ROS-scavenging in *Nicotiana tabacum* (Zhao et al., 2018); and *PebHLH35* improves plant tolerance to drought by regulating stomatal density and stomatal pore size (Dong et al., 2014).

Apples constitute an important part of the daily human diet and are one of the best-loved fruit in many parts of the world. The trees are most affected by drought stress, which significantly decreases fruit yield and quality. Thus, they have developed a number of sophisticated mechanisms to adapt to the surrounding conditions. Recently, some bHLH TFs have been involved in response to drought stress in plants (Singh and Laxmi, 2015). However, only a few apple bHLH genes have been functioned in drought response. Mao et al. (2017) identified 188 MdbHLH proteins in apple, and some of them regulated responses to drought stress. Here, we further identified a bHLH gene, *MdbHLH130*, which is highly induced by dehydration treatment, and selected it for further study. Transgenic apple calli overexpressing *MdbHLH130* displayed greater resistance to PEG6000 treatment and were more sensitive to the suppression of *MdbHLH130* compared with that of the wild type (WT). The overexpression of *MdbHLH130* in tobacco increased water deficit tolerance, reduced electrolyte leakage and malondialdehyde (MDA) levels, elevated antioxidant enzyme activity, and increased the expression of ROS-scavenging genes and stress-related genes compared with the WT plants. Moreover, *MdbHLH130*-overexpressing plants showed decrease sensitivity to oxidative stress. Overall, these data might provide new insights into the mechanism of bHLH proteins in response to water deficit stress.

## Materials and Methods

### Plant Materials and Treatments

Apple (*Malus × domestic* ‘Royal Gala’) seedlings were grown at 23°C under 8/16-h dark/light photoperiod. The method of dehydration treatment was performed according to Liao et al., 2017. The seedlings were transferred to filter paper and dried at 23°C for the indicated times (0, 1, 3, 6, 12, and 24 h), and then immediately frozen in liquid nitrogen and stored at −80°C until RNA extraction.

‘Orin’ apple calli (wild-type) were used for genetic transformation in this study. The calli were cultivated on medium as previously described (Zhao et al., 2016).

Tobacco cultivars (*N. tabacum* L. “NC89”) were used for genetic transformation. The seeds were surface-sterilized with 2.6% NaClO for 10 min and then germinated on 1/2 MS medium containing 2.5% sucrose and 1.0% agar at 25 ± 1°C with a 16-h light/8-h dark photoperiod.

### Real-Time Quantitative PCR

Total RNA was extracted from the apple seedlings, apple calli, and tobacco whole seedlings using TRIzol reagent (Invitrogen, Carlsbad, CA, USA) following the manufacturer’s instructions, and then reverse transcripts were synthesized with a PrimeScript 1^st^ Strand cDNA Synthesis Kit (TaKaRa, Dalian, China). Real-time quantitative PCR was carried out on an Applied Biosystem 7500 Real-Time PCR System (Applied Biosystems, Foster City, CA, USA) using the SYBR Premix Ex Taq (TaKaRa, Dalian, China) in a 20-μl volume. The PCR reaction conditions were as follows: 95°C for 30 s, followed by 40 cycles of 95°C for 5 s and 60°C for 34 s. The *MdActin* (GenBank: CN938023) was used as an internal reference for apple, while *NtActin* (GenBank: U60495) was used for tobacco. Relative gene expression analyses were conducted using the cycle threshold (Ct) 2^−Δ;Δ;CT^ method (Livak and Schmittgen, 2001). Each sample at each time point was performed in three independent replicates. The primer sequences in this study are shown in **Table S1**.

### Plasmid Construction and Genetic Transformation

To generate the *P_MdbHLH130_::GUS* construct, a 2.0 kb promoter fragment was amplified and inserted into the pCXGUS-P vector containing the GUS reporter gene. For the overexpression vector, the full-length cDNA of *MdbHLH130* was cloned into pRI-GFP vectors under the control of a CaMV 35S promoter. For the antisense suppression vector, a 280-bp specific fragment of *MdbHLH130* was amplified and linked into the pRI vector. Then, the recombinant plasmid *P_MdbHLH130_::GUS*, pRI-*MdbHLH130*-GFP and pRI-*MdbHLH130*-Anti were introduced into the *A. tumefaciens* strain EHA105. The primer sequences are shown in **Table S2**.

Transgenic apple calli were obtained as described previously (Zhao et al., 2016). ‘Orin’ apple calli were immersed into *Agrobacterium* suspension cultures for 10-15 min. Then, the calli were co-cultivated in MS medium with 1.5 mg^−1^ L 2,4-D and 0.4 mg^−1^ L 6-BA at 23 ± 1°C in the dark for 2–3 days. After co-cultivation, the calli were transferred to the screening medium containing MS + 1.5 mg^−1^ L 2,4-D + 0.4 mg^−1^ L 6-BA + 100 mg^−1^ L kanamycin + 250 mg^−1^ L carbenicillin to obtain transgenic calli.

For transgenic tobacco, the *A. tumefaciens* strain EHA105 containing binary constructs were transformed into tobacco by leaf disc transformation (Horsch et al., 1985). The leaves were cut into small strips and immersed into *A. tumefaciens* suspension cultures for 15 min. Then, the pieces were dried with sterile filter paper and screened onto selection medium (containing 100 mg^−1^ L kanamycin). T3 seedlings from three independent transgenic lines were used in further investigations.

### GUS Staining and Activity Analyses

GUS staining of *Pro_MdbHLH130_::GUS* transgenic apple calli and the WT was performed according to Jefferson et al. (1987). The apple calli were transferred to a filter paper and dried at 23°C for 4 h and then immersed into GUS staining buffer at 37°C in the dark overnight. After staining, the samples were de-stained and photographed.

GUS activity was detected as described previously (Jefferson et al., 1987). The total proteins from the samples were extracted with extraction buffer and reacted with 4-MUG (Sigma-Aldrich, St. Louis, MO, USA) at 37°C. The GUS activity was determined using a VersaFlour spectrofluorometer (Bio Rad Laboratory Inc., Hercules, CA) at an Ex = 365 nm and Em = 450 nm.

### Subcellular Localization and Transactivation Assay

The construct *35S::MdbHLH130-GFP* (pRI-*MdbHLH130*-GFP) was transferred into the *A. tumefaciens* GV3101 strain. Then, the *Agrobacterium*-harboring *35S::MdbHLH130-GFP* construct was infiltrated into the epidermal cells of *Nicotiana benthamiana* leaves at 24°C for 2–3 days. 4′,6-diamidino-2-phenylindole (DAPI) was used to stain the nuclei, and the fluorescence was photographed with a confocal laser scanning microscope (E_x_ = 488 nm by argon laser, E_m_ = 505–530 nm by BP filter) (Zeiss LSM510 Meta, Germany).

The open reading frames (ORFs) of *MdbHLH130* were cloned into the pGBKT7 vector (Clontech) to generate pGBKT7-*MdbHLH130*. Then, the pGBKT7-*MdbHLH130* + pGADT7 was transformed into the yeast strain Y2H Gold. The transformants were spotted on SD/-Trp and SD/-Trp/-His/-Ade medium.

### Protein Extraction and Western Blotting

Samples of tobacco whole seedlings were ground in a protein extraction buffer. The proteins were separated by SDS-PAGE gel and then transferred onto polyvinylidene fluoride (PVDF) membrane (Roche, USA). The signals were visualized using the ECL plus kit (Millipore, Bedford, MA) and the MdbHLH130 protein levels were detected with anti-GFP antibody as described previously (Burnette, 1981). ACTIN served as a protein-loading control.

### Stress Tolerance Assay

For the PEG 6000 treatment and germination assay, the seeds of transgenic lines and WT were sterilized by 2.6% NaClO and planted on 1/2 MS medium with or without 10% PEG6000, and then the germination rates were measured daily (Quan et al., 2004). For the root length assay, the seeds were germinated on 1/2 MS medium for 10 d. Seedlings with the same root length were then transferred to 1/2 MS medium supplemented with 10% PEG6000 and placed upright in the chamber. The root lengths were measured after the treatment.

For the drought treatment, 35-day-old *MdbHLH130*-overexpression and WT plants were grown in a greenhouse and adequately watered. Subsequently, water was withheld for 15 d, and the survival rates were recorded after re-watering for 7 d. To estimate the water loss, fully expanded leaves were detached from 35-day-old plants and weighed immediately. Then, the leaves were placed on filter paper in the ambient environment and weighed at the designated times, and the water loss rate was calculated relative to that of the initial fresh weights.

For the oxidative stress treatment, the leaf discs were detached from the fully expanded leaves of 35-day-old plants and incubated in a methyl viologen (MV) solution (0 or 100 μM) for 48 h, and then the Chl contents were measured. All the results are based on the average of three independent biological replicates.

### Stomatal Aperture Ratio Analysis

Leaves of the WT and transgenic lines were incubated in an opening solution (10 mM KCl, 50 mM CaCl_2_, and 10 mM MES-Tris, pH 6.15) for 4 h under light conditions. To observe the stomatal aperture ratio under dehydration stress conditions, the leaves were placed on filter paper for 4 h. To investigate the ABA response, the leaves were treated with 10 µM ABA solution for 4 h. Subsequently, the stomata were imaged using a microscope (Olympus ix71, Tokyo, Japan). The widths and lengths of the stomata were determined with ImageJ software. At least 100 stomata measurements were performed for each treatment.

### ROS Staining and Physiological Measurements

ROS levels were monitored using the fluorescent probe 2′,7′-dichlorodihydrofluorescein diacetate (DCFH2-DA, Sigma-Aldrich, USA). The leaves were treated with 10 µM DCFH2-DA for 30 min and then washed with distilled H_2_O. The images were obtained using the confocal microscope (E_x_ = 488 nm; E_m_ = 522 nm) (Leica Microsystems, Wetzlar, Germany). Fluorescence intensity of leaves was analyzed using ImageJ software.

The electrolyte leakage (EL) measurement in leaves was examined according to the method of Lurie and Ben-Arie (1983). The leaves were placed in distilled water for 3–4 h at room temperature and measured with electric conductivity meter (E1). Then the samples were boiled for 20 min and cooled to room temperature, the electrolyte conductivity (E2) was measured, and the Electrolyte leakage (%) = 100 × E1/E2. The malondialdehyde (MDA) contents were measured using the thiobarbituric acid-based method with a MDA assay kit (A003-3, Jiancheng, Nanjing, China). For the total chlorophyll content (a + b), the young leaves were collected and immersed in 80% acetone and then centrifuged at 10,000 g for 5 min. The total chlorophyll concentrations were measured from the spectroscopy absorbance measurements at 663, 645, and 480 nm.

For the enzyme assays, the SOD activity was measured following the manufacturer’s instructions of the SOD Detection Kit (A001, Jiancheng, Nanjing, China). The CAT activity was measured using a CAT Detection Kit by detecting the degradation of H_2_O_2_ at 405 nm (A007, Jiancheng, Nanjing, China). The POD activity was detected in the leaves according to POD Detection Kit for plant (A084-3, Jiancheng, Nanjing, China). Each assay was replicated at least three times per sample.

### Statistical Analysis

The SPSS 16.0 software (SPSS Inc., Chicago, IL, USA) was used for data analyses. Experimental data were analyzed with Student’s *t*-test, taking P < 0.01 (*) as significant. All experiments were repeated at least three replicates, and sample variability is given as the standard deviation (SD) of the mean.

## Results

### Phylogenetic Tree and Sequence Analysis of MdbHLH130

**Figure 1:**
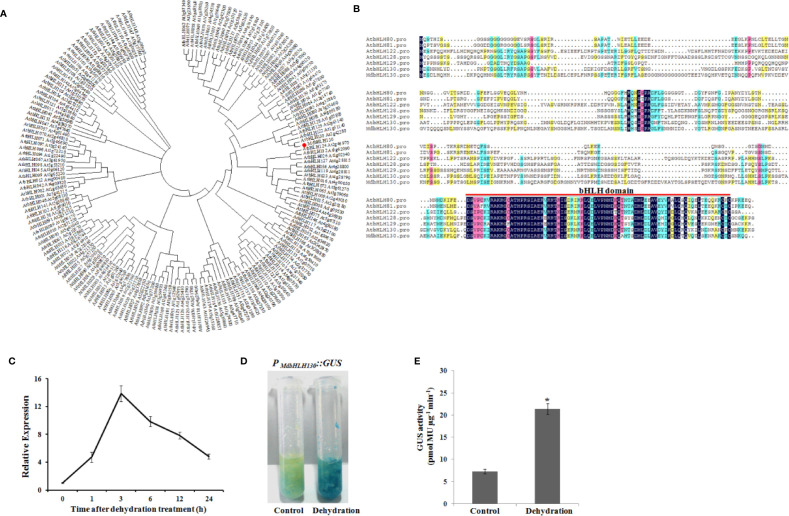
Phylogenetic tree, sequence alignment and transcript levels of the *MdbHLH130*. **(A)** Phylogenetic analysis of bHLH proteins between 166 AtbHLH proteins and the MdbHLH130 protein *via* MEGA4.0 using the neighbor-joining method. MdbHLH130 is in the red box. **(B)** Multiple sequence alignment of the MdbHLH130 protein with its known *Arabidopsis* homologs. Identical amino acids are shaded in black. The conserved bHLH motif is indicated with a red line. **(C)** qRT-PCR analysis of *MdbHLH130* observed in 30-day-old apple seedlings after the dehydration treatment applied for 0, 1, 3, 6, 12, and 24 h. Data are the means ± SD of three independent biological replicates. **(D)** and **(E)** GUS staining and activity analysis of *P_MdbHLH130_::GUS* transgenic apple calli after the dehydration treatment. Error bars indicate the means ± SD from three independent biological replicates. Asterisks indicate significant differences relative to the control (*P < 0.01).

Studies have reported that bHLH TFs have important functions in the regulation of plant stress responses. Previous studies have shown that a bHLH TF (*MDP0000581816*) is upregulated by drought stress treatment (Mao et al., 2017; Benny et al., 2019), suggesting that it may be involved in drought stress tolerance; thus, it was selected for further study. The phylogenetic tree of all bHLH genes of apple has been analyzed by Mao et al. (2017). The MDP0000581816 protein clustered within the same clade as MDP0000307685 (similarity 75.37%), so the *MDP0000581816* gene is not duplicated. The open reading frame of *MDP0000581816* is 1236 bp in length and encodes proteins with 411 amino acids. The phylogenetic tree showed that MDP0000581816 shared most sequence similarities with the uncharacterized *Arabidopsis* gene AtbHLH130 (AT2G42280), which is named MdbHLH130 (**Figure 1A**). Multiple sequence alignment showed that MdbHLH130 had a single bHLH conserved domain in the C-terminal (**Figure 1B**).

### *MdbHLH130* Is Induced by Dehydration Stress

The expression pattern of *MdbHLH130* was examined in response to dehydration stress *via* a quantitative real-time (qRT) PCR analysis, and the results showed that *MdbHLH130* expression levels were greatly induced and gradually reached a peak at 3 h (12.6-fold) and then decreased (**Figure 1C**). To further confirm the expression patterns of *MdbHLH130*, the promoter region (2.0 kb) was cloned and fused to a *β*-glucuronidase (GUS) reporter gene to achieve *P_MdbHLH130_::GUS* transgenic apple calli. Consistent with the qRT-PCR analysis results, the GUS staining and activity results revealed that *P_MdbHLH130_::GUS* transgenic apple calli had higher GUS activity compared with the control under dehydration stress treatment (**Figures 1D, E**). These results indicated that the expression of *MdbHLH130* is induced by dehydration stress.

### MdbHLH130 Is a Nuclear Protein That Function as a TF

**Figure 2:**
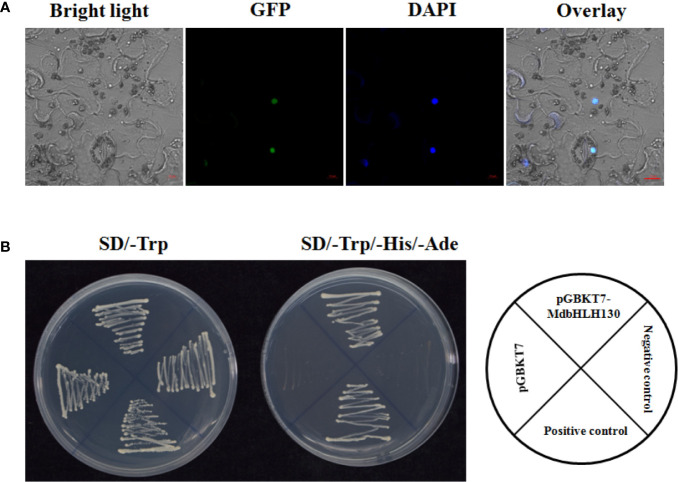
Subcellular localization and transcriptional activity of the MdbHLH130 protein. **(A)** Subcellular localization of MdbHLH130. The *35S::MdbHLH130-GFP* fusion proteins were transiently transferred into epidermal cells of *N. benthamiana* leaves. Green fluorescence was visualized using confocal microscopy. Scale bar, 10 μm. **(B)** Transactivation assay of MdbHLH130 in yeast. The yeast cells transformed with different constructs on SD/-Trp or SD/-Trp/-His/-Ade medium for 3–5 days.

The bioinformatic prediction analysis implied that MdbHLH130 might be targeted to the nucleus (PredictNLS software). To determine the subcellular localization of MdbHLH130, the CDS region of *MdbHLH130* was fused with the green fluorescent protein (GFP) reporter gene under the control of the CaMV 35S promoter. Because the *Agrobacterium-*harboring *35S::MdbHLH130-GFP* construct had difficulty infiltrating into the epidermal cells of apple leaves, the sub-localization of apple genes were usually done in *Nicotiana benthamiana* leaves. The construct was then transiently injected into the epidermal cells in *N. benthamiana* leaves using *A. tumefaciens*-mediated transformation. After 2–3 days, the GFP signal was detected only in the nucleus of epidermal cells (**Figure 2A**), indicating that MdbHLH130 was localized to the nucleus. Nuclear localization was confirmed by staining with DAPI.

bHLH TFs have been reported to possess transcriptional activity (Toledo-Ortiz et al., 2003). To determine whether MdbHLH130 had transcriptional activity, the full-length MdbHLH130 coding sequence (CDS) was fused with a GAL4 DNA-binding domain (GDBD) in the yeast expression vector pGBKT7. The different combinations were transformed separately into the yeast strain Y2H Gold. The different transformants were transformed, and all grew well on synthetic dextrose synthetic dropout (SD)/-Trp medium. However, the yeast cells transformed with the control vector did not survive on selective SD/-Trp/-His/-Ade media. In contrast, yeast cells harboring pGBKT7-MdbHLH130 grew normally on the same medium (**Figure 2B**), indicating that MdbHLH130 exhibited transcriptional activity in yeast cells.

### Stress Tolerance Assay of *MdbHLH130* in Transgenic Apple Calli

**Figure 3:**
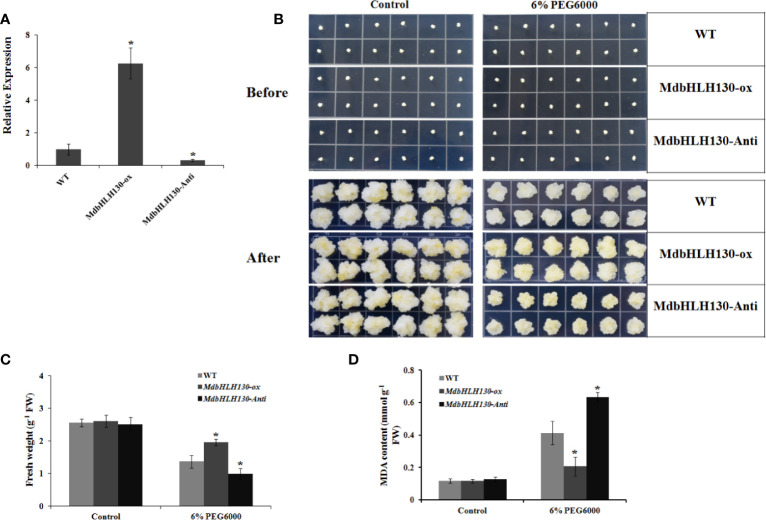
Effect of *MdbHLH130* on PEG6000 tolerance in transgenic apple calli. **(A)** Expression analysis of *MdbHLH130* in WT, *MdbHLH130-ox* and *MdbHLH130-Anti* transgenic calli by RT-PCR. The results were normalized using the internal control *MdActin*. Data are expressed as the mean ± SD as determined from three independent biological replicates. **(B)** Growth phenotypes of WT, *MdbHLH130-ox* and *MdbHLH130-Anti* transgenic apple calli. The WT and transgenic apple calli were grown on medium at 23°C for 7 days and then treated with 6% PEG6000 for another 20 days. **(C, D)** Fresh weights and MDA contents of WT, *MdbHLH130-ox* and *MdbHLH130-Anti* transgenic apple calli under the control or PEG6000 treatment conditions for another 20 days. Error bars represent the means ± SD taken from three independent biological replicates. Asterisks indicate significant differences relative to the WT (*P < 0.01).

Because it is difficult to obtain transgenic apple plants using the apple calli “Orin”, so the apple calli were thereafter used for function analysis. As a model system, the apple transgenic calli were used to characterize the function of some genes in modulating stresses tolerance (Zhao et al., 2016; Ma et al., 2019; Ren et al., 2019). To characterize the functions of *MdbHLH130*, the ORF sequence and specific cDNA fragments were cloned and fused into the PRI vector to generate *35S::MdbHLH130* (*MdbHLH130-ox*) and *35S::MdbHLH130-Anti* (*MdbHLH130-Anti*) constructs, respectively. Then, the constructs were transformed into apple calli (‘Orin’). qRT-PCR analysis indicated that the *MdbHLH130-ox* line had higher transcript levels relative to the WT control while the *MdbHLH130-Anti* calli produced lower levels (**Figure 3A**), indicating that transgenic apple calli were obtained. Subsequently, the 10-day-old *MdbHLH130-ox*, *MdbHLH130-Anti*, and WT calli were grown on MS medium with or without 6% PEG6000 for another 20 days. Compared with the control, *MdbHLH130-ox* calli exhibited faster growth than the WT control, while the *MdbHLH130-Anti* calli had the opposite phenotype (**Figures 3B, C**). Consistent with the phenotype, the MDA content levels in the *MdbHLH130-ox* calli were lower than that of the WT lines, while the *MdbHLH130-Anti* calli showed increased levels (**Figure 3D**). Overall, *MdbHLH130* enhances the tolerance to PEG6000 in transgenic apple calli.

Next, we examined the transcript abundances of ROS-scavenging and stress-related genes. qRT-PCR results indicated that the gene expression in *MdbHLH130-OX* lines was slightly higher than in the *MdbHLH130-Anti* lines and wild-type under control conditions. However, the transcript levels were significantly higher in the *MdbHLH130-OX* lines than in wild-type, whereas suppression of *MdbHLH130* had the opposite results (**Supplementary Figure S1**).

### Overexpression of *MdbHLH130* in Tobacco Enhances Water Deficit Tolerance

Transgenic tobacco plants overexpressing *MdbHLH130* were generated to further investigate the role of *MdbHLH130* in water deficit stress tolerance. A total of eight independent transgenic lines were obtained using PCR and western blotting analyses (**Supplementary Figures S2A–C**). Three T3 homozygous lines (L1, L2, and L3) were selected, and they had higher *MdbHLH130* expression levels; thus, they were selected for further analyses using semi-quantitative RT-PCR and western blotting (**Supplementary Figures S3A, B**).

**Figure 4:**
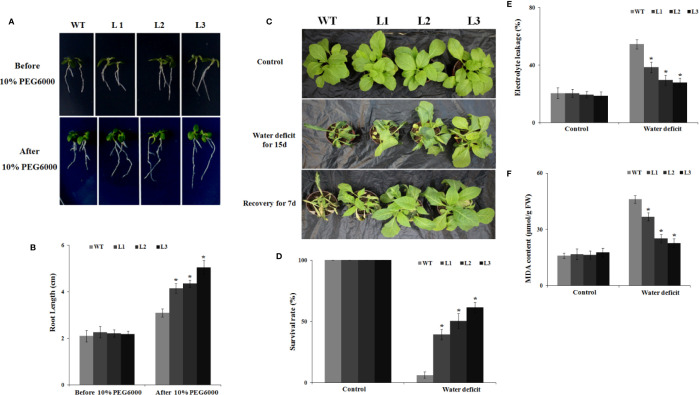
Water deficit stress tolerance to tobacco plants overexpressing *MdbHLH130*. **(A, B)** Primary root growth of the WT and *MdbHLH130-ox* (L1, L2, and L3) seedlings before and after 10% PEG6000 treatment. The seedlings were grown on 1/2 MS medium supplemented with 10% PEG6000 and placed upright in the chamber for 8 days. **(C)** Water deficit stress phenotypes of the WT and *MdbHLH130* transgenic lines. The WT and three transgenic tobacco lines (L1, L2, and L3) were grown at 24°C and then withheld water for 15 days, and photos were taken 7 days after the plants were re-watered. **(D, E)** Statistical analysis of the survival rates **(D)**, electrolyte leakage **(E)** and MDA contents **(F)** in the WT and transgenic lines after water deficit treatment. FW, Fresh weight. Error bars represent the means ± SD of more than 30 plants from three independent biological replicates. Asterisks indicate significant differences relative to the WT (*P < 0.01).

First, the germination rates of the WT and *MdbHLH130-ox* plants on MS medium with or without 10% PEG6000 were explored. On MS medium, the *MdbHLH130-ox* lines and WT plants showed similar germination rates (**Supplementary Figure S4**). However, supplementation with 10% PEG6000 significantly inhibited the germination rates of the WT but had a limited effect on the *MdbHLH130-ox* transgenic plants (**Supplementary Figure S4**). To further evaluate the effects of drought stress on the transgenic plants, 10-day-old WT and *MdbHLH130-ox* seedlings were placed on MS medium containing 10% PEG6000 for 8 days. The root elongation was significantly higher for the *MdbHLH130-ox* (3.7 to 4.6 cm) transgenic plants than for the WT (3.1 cm) plants (**Figures 4A, B**), indicating that *MdbHLH130* is involved in the response to PEG6000 treatment at the germination and root elongation stages.

Subsequently, 35-day-old seedlings of the WT and transgenic plants were exposed to water deficit for 15 days in soil. Most of the WT plants wilted, whereas the *MdbHLH130-ox* transgenic plants remained turgid and green (**Figure 4C**). Moreover, 45–63% of the *MdbHLH130-ox* transgenic plants survived after re-watering, which was significantly higher than for the WT (11%) plants (**Figure 4D**). These results suggested that *MdbHLH130-ox* plants may have depleted their water resources more slowly than the WT transgenic plants and thus grew well. Additional indicators of stress are the malondialdehyde (MDA) content and electrolyte leakage (EL), which can reflect the degree of damage to cell membranes (Moore and Roberts, 1998). MDA and EL accumulation were detected in the transgenic lines and WT under water deficit stress. The transgenic lines exhibited significantly less MDA and EL accumulation compared to the WT (**Figures 4E, F**), confirming that the overexpression of *MdbHLH130* reduced the damage and maintained the membrane integrity during water deficit stress. In addition, the total chlorophyll levels in the leaves of the WT plants (0.22 mg/g FW) were also lower than those of the transgenic plants (0.37–0.53 mg/g FW) after the water deficit treatment (**Supplementary Figure S5A**). Consistent with the total chlorophyll levels, the P_N_ and Fv/Fm values were decreased in both the WT and transgenic plants under water deficit stress; however, their values remained higher in the transgenic plants compared with the WT (**Supplementary Figures S5B, C**).

**Figure 5:**
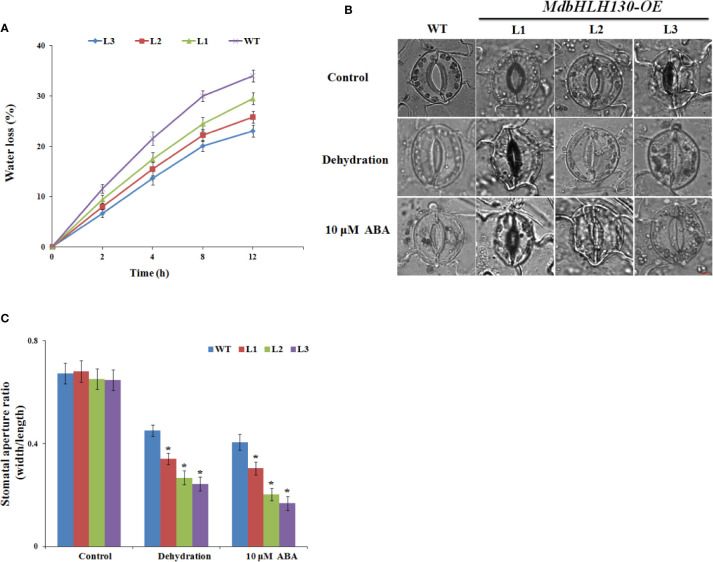
Water loss rate and stomatal aperture ratios from WT and *MdbHLH130-ox* plants after treatments. **(A)** Water loss of the detached leaves from the WT and *MdbHLH130-ox* plants. **(B, C)** Images of stomata and measurement of stomatal aperture ratios (width/length ratio) of the WT and *MdbHLH130-ox* plant leaves in response to dehydration or 10 μM ABA. Scale bar, 10 μm. Error bars represent the means ± SD of 100 stomata from three independent biological replicates. Asterisks indicate significant differences between *MdbHLH130-ox* plants and WT (*P < 0.01).

To investigate whether there was a difference in water loss rate, we measured the water loss rates from the leaves of the WT and *MdbHLH130-ox* plants under normal and drought conditions. As shown in **Figure 5A**, the water loss rate of the leaves from the transgenic *MdbHLH130-ox* plants was much lower compared with that of the WT plants after the dehydration treatment, indicating that *MdbHLH130* overexpression in tobacco leads to superior water status. Consistent with these results, the stomatal aperture ratio indices of the *MdbHLH130-ox* plants decreased to 0.32–0.24, which was smaller than that of the WT after 4 h of dehydration (**Figures 5B, C**). A previous study reported that ABA-mediated stomatal closure is crucial to reducing transpiration under drought stress (Castilhos et al., 2014). We checked the leaf stomatal aperture ratios in the presence of exogenous ABA, and the stomatal aperture ratio index was 0.40 for the WT plants and 0.28–0.17 for the *MdbHLH130-ox* plants (**Figures 5B, C**). These results suggested that *MdbHLH130* improved the water deficit tolerance at least partly by promoting the plants’ ability to close their stomata and reduce transpiration.

Overall, these results indicate that *MdbHLH130* functions as a positive regulator in plant responses to water deficit stress.

### ROS Accumulation and Antioxidant Enzyme Activities in *MdbHLH130*-Overexpressing Plants Under Water Deficit Stress Conditions

**Figure 6:**
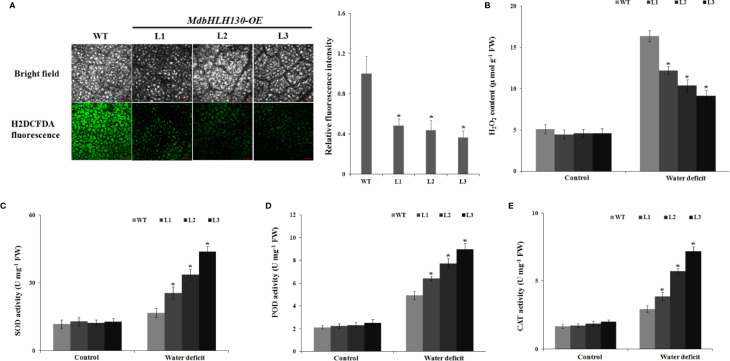
ROS accumulation and activity of antioxidant enzymes in *MdbHLH130-ox* plants and WT after water deficit treatment. **(A)** Fluorescence detection and quantification of ROS by H2DCFDA staining in *MdbHLH130-ox* plants and WT leaves after water deficit treatment. Bar = 250 µm. **(B–E)** Statistical analysis of the H_2_O_2_ content **(B)**, SOD activity **(C)**, POD activity **(D)**, and CAT activity **(E)** in the WT and three *MdbHLH130-ox* plants after water deficit treatment. Error bars represent the means ± SD taken from three independent biological replicates. Asterisks indicate significant differences relative to the WT (*P < 0.01).

Water deficit leads to the production of ROS, and excess ROS levels cause damage to cell growth following oxidative stress (Choudhury et al., 2017). To test whether *MdbHLH130* regulates ROS levels in response to water deficit stress, leaves from the WT plants and *MdbHLH130-ox* plants were stained with 2′,7′-dichlorofluorescin diacetate (H2DCFDA) after water deficit treatment for 10 days. ROS staining in *MdbHLH130-ox* plants was lower than that of the WT plants under water deficit treatment (**Figure 6A**), showing that the WT plants accumulated more ROS. Consistent with these results, the H_2_O_2_ content levels for the transgenic plants were lower than those of the WT plants under water deficit stress (**Figure 6B**).

Superoxide dismutase (SOD), catalase (CAT), and peroxidase (POD) are important antioxidant enzymes responsible for scavenging ROS in response to various stresses (Choudhury et al., 2017; Mittler, 2017). Thus, we confirmed the activities of these enzymes. Under normal conditions, the SOD, POD, and CAT activities in the transgenic lines showed no differences compared with that of the WT (**Figures 6C–E**). However, after the water deficit stress treatment, the transgenic lines presented significantly higher SOD, POD, and CAT activities than the WT (**Figures 6C–E**), which is consistent with ROS staining. These results suggest that the overexpression of *MdbHLH130* reduced ROS accumulation under water deficit stress by enhancing the activities of ROS-scavenging enzymes.

### MdbHLH130 *Positively Regulates Genes Related to ROS Scavenging and* Stress Responses Under Water Deficit Stress Conditions

**Figure 7:**
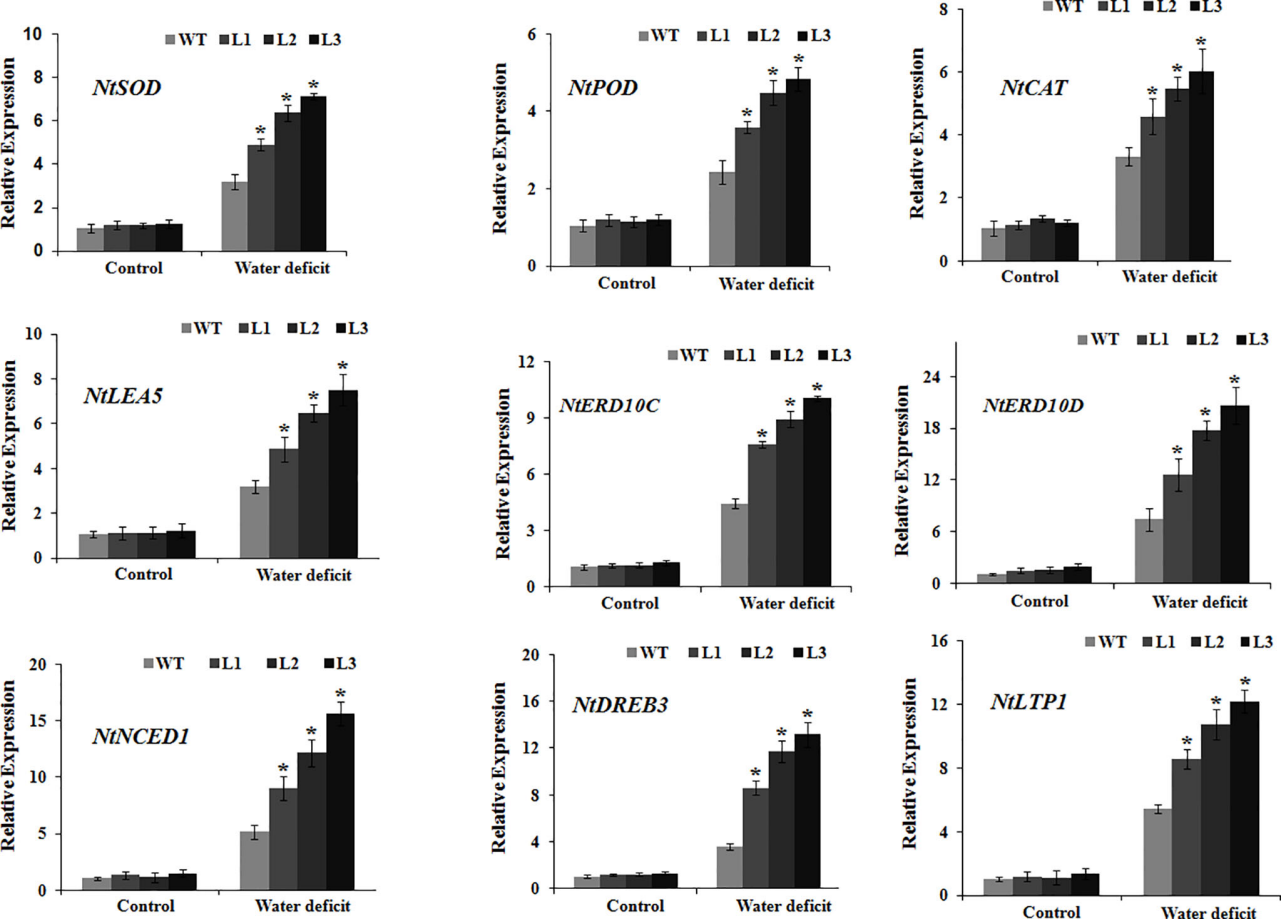
Expression of the genes involved in ROS scavenging and stress responses in the WT and transgenic plants. The expression levels were analyzed by qRT-PCR in tobacco seedlings under the control or water deficit treatment conditions. Error bars represent the means ± SD taken from three independent biological replicates. Asterisks indicate significant differences from the WT (*P < 0.01).

To obtain additional insights into the molecular mechanisms underlying the *MdbHLH130*-mediated signaling pathway in water deficit resistance, we examined the transcript abundance of genes related to ROS scavenging (*NtSOD*, *NtPOD*, and *NtCAT*) and stress responses (*NtDREB3*, *NtERD10C*, *NtERD10D*, *NtNCED1*, *NtLEA5*, and *NtLTP1*) by qRT-PCR. Under normal conditions, the mRNA abundance of genes related to ROS scavenging and stress responses did not differ between the WT and *MdbHLH130-ox* plants (**Figure 7**). Upon exposure to water deficit conditions, however, the transcript levels of these genes were significantly higher in the *MdbHLH130-ox* plants compared with the WT plants (**Figure 7**). These data suggest that *MdbHLH130* increases water deficit tolerance in tobacco plants by upregulating the transcript levels of genes related to ROS scavenging and stress responses.

### *MdbHLH130* Overexpression Enhances Oxidative Stress Tolerance

**Figure 8:**
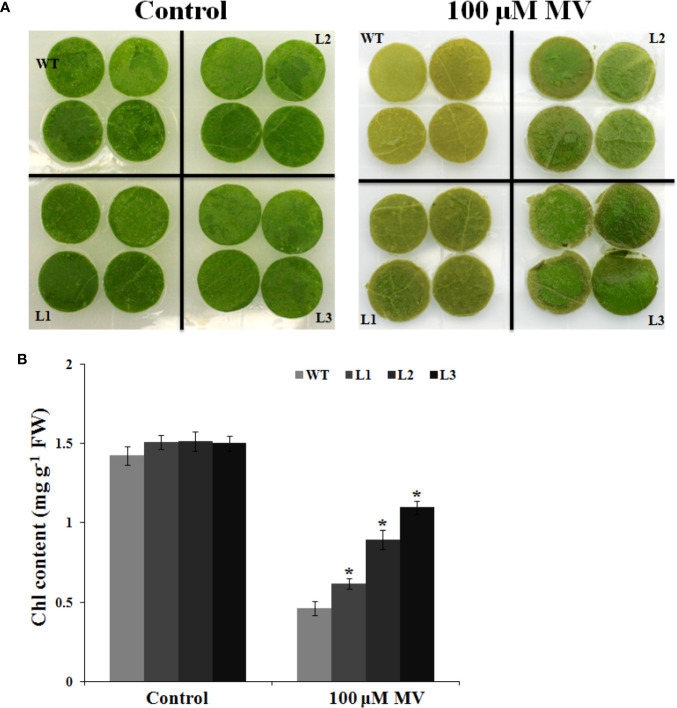
Oxidative stress tolerance to *MdbHLH130-ox* plants. **(A)** Phenotype of the leaf discs from the WT and three *MdbHLH130-ox* plants after incubation with or without 100 μM MV solution for 48 h. **(B)** Relative total chlorophyll content in the leaf discs after 100 μM MV treatment. Three independent biological replicates were performed. Vertical bars refer to means ± SD of at least three independent biological replicates. Asterisks indicate a significant difference between the WT and *MdbHLH130-ox* lines (*P < 0.01).

To assess the role of *MdbHLH130* in regulating antioxidant mechanisms, we investigated the relationship between *MdbHLH130* and oxidative stress. Methyl viologen (MV) is a herbicide that causes Chl degradation and cell membrane leakage through ROS production (Kurepa et al., 1998). Leaf discs were incubated in distilled water or 100 μM methyl viologen (MV) solution. There were no conspicuous differences between the WT and transgenic lines incubated in distilled water, whereas after 100 μM MV treatment, the WT exhibited more browned and necrotic leaf discs than did the transgenic lines (**Figure 8A**). The Chl content was higher in the transgenic lines than in the WT (**Figure 8B**). These data indicate that ectopic expression of *MdbHLH130* in tobacco decreased the sensitivity to oxidative stress.

## Discussion

In nature, plant growth and development and crop productivity are inevitably affected by many abiotic stresses, such as drought stress (Bray et al., 2000; Shinozaki and Yamaguchi-Shinozaki, 2000; Zhu, 2016). To survive, plants have evolved a wide range of mechanisms to respond to drought stress (Singh and Laxmi, 2015). Among the numerous stress-related genes, transcription factor (TF) genes are modulated in plants under drought stress to help the plants maintain normal growth and development (Zhu, 2016). bHLH proteins are the second largest subfamily of TFs in plants and participate in various biological processes, including drought stress response, by binding to the G-box/E-box *cis*-elements in the promoter regions of its target genes (Robinson and Lopes, 2000; Toledo-Ortiz et al., 2003; Song et al., 2016). However, only a few apple bHLH genes have been reported to play essential roles in drought stress tolerance. In this study, we isolated a bHLH gene named *MdbHLH130* from apple (**Figure 1**). The overexpression of *MdbHLH130* in apple calli and tobacco transgenic plants showed enhanced resistance to water stress (**Figures 3**, **4**), indicating that *MdbHLH130* is a positive regulatory factor involved in the water stress tolerance of plants.

Previous studies showed that bHLH genes played important roles in response to abiotic stresses (Seo et al., 2011). For instance, the *PtrbHLH* gene is upregulated by various stresses, particularly cold stress (Huang et al., 2013); and the *OrbHLH2* gene is upregulated by salt and osmotic stress but not by cold stress (Zhou et al., 2009). In our work, the *MdbHLH130* transcript level was induced by dehydration treatment (**Figures 1C–E**), indicating that it possibly plays a role in regulating the response to water stress. bHLH TFs are involved in several processes, including stomatal development, root hair formation, root meristem size, and hormone metabolism in response to drought stress (Abe et al., 2003; Pillitteri and Torii, 2007; Makkena and Lamb, 2013; Castilhos et al., 2014). Stomata represent a key determinant for transpirational water loss, and stomatal closure results in reduced water loss, and this behavior is critical for maintaining a high water potential under drought stress conditions in plants (Nilson and Assmann, 2007). In *Arabidopsis*, the ABA levels were markedly induced under drought conditions, resulting in stomatal closure (Hu et al., 2013). Drought stress has been reported to trigger the ABA-dependent signaling pathway (Zhu, 2002). Certain bHLH genes have been reported to be induced in response to drought stress dependent on the ABA-mediated pathway. For example, *AtMYC2* functions as a transcriptional activator in the ABA-mediated drought stress signaling pathway (Abe et al., 2003). Overexpression of the *bHLH122* gene increased the resistance of transgenic plants to drought and osmotic stresses by increasing the cellular ABA levels (Liu et al., 2014). In this study, the stomatal aperture ratio indices of the *MdbHLH130* transgenic tobacco lines were lower than those observed of the WT plants after the dehydration treatment and ABA treatments (**Figure 5**). In accordance with these results, the leaf water loss rate of the transgenic tobacco plants was low (**Figure 5**). Moreover, the gene encoding *NtNCED1*, a key enzyme of ABA biosynthesis, is a known participant in ABA-mediated responses (Shinozaki et al., 2003; Hu et al., 2013). The expression of *NtNCED1* was upregulated in the *MdbHLH130*-overexpressing plants compared to the WT plants, implying that ABA biosynthesis may be promoted in transgenic plants (**Figure 7**). Therefore, these results indicate that *MdbHLH130* functions as a positive regulator of water deficit stress responses by partly involving ABA-mediated stomatal aperture ratio and transpiration. In addition, photosynthesis is an essential process for maintaining plant growth and development, and total chlorophyll fluorescence parameters (*e.g.*, Fv/Fm) represent a rapid and accurate approach for detecting and quantifying water stress tolerance in plants (Percival and Sheriffs, 2002; Anjum et al., 2011). Under the water deficit stress condition, the transgenic plants exhibited higher survival rates and better total chlorophyll contents based on phenotypic observations, suggesting that *MdbHLH130* conferred tolerance to water deficit stress by regulating different pathways (**Figure S5**).

EL is an indicator of the severity of a membrane injury, and MDA is a product of oxidative attack on membrane functionality and integrity (Moore and Roberts, 1998). Under water deficit stress conditions, the transgenic tobacco plants exhibited decreased IL and MDA contents (**Figures 4E, F**). *NtERD10C*, *NtERD10D*, and *NtLEA5* encode the late embryogenesis abundant (LEA) proteins that protect cellular and macromolecular structures during dehydration tolerance (Hundertmark and Hincha, 2008). The higher expression of *NtERD10C*, *NtERD10D*, and *NtLEA5* in transgenic tobacco plants resulted in greater water retention and less membrane damage compared to the WT plants under water deficit stress (**Figures 4**, **7**). ROS, which mainly include O^2−^ and H_2_O_2_, cause damage to cell growth by oxidizing proteins, lipids, and DNA or acting as signal molecules that mediate tolerance to various stresses (Choudhury et al., 2017; Mittler, 2017). Previous studies have shown that the overexpression of *PtrbHLH* or *AtbHLH112* increases SOD or POD activity and reduces ROS accumulation under stress conditions (Huang et al., 2013). Given the importance of ROS in stress tolerance, we investigated whether ROS-scavenging ability is regulated by *MdbHLH130*. The staining and measurements results revealed that ROS accumulated at lower levels in the *MdbHLH130-ox* tobacco plants compared with the WT plants (**Figures 6A, B**). Furthermore, the activities of three important ROS-scavenging enzymes were higher in the *MdbHLH130*-expressing tobacco plants (**Figures 6C–E**). Moreover, the transcripts of the ROS-scavenging genes were positively correlated with the transcript levels of *MdbHLH130* (**Figure 7**). In addition, the *MdbHLH130-ox* transgenic plants were damaged to a less serious degree under the MV treatment, suggesting that they were more resistant to oxidative stresses than the WT (**Figure 8**). Taken together, these results indicate that *MdbHLH130* might function to mediate the transcriptional upregulation of ROS-scavenging genes to enhance the ROS-scavenging ability and improved water deficit stress tolerance.

Plants have evolved a variety of mechanisms to improve drought tolerance (Miller et al., 2010). Dehydration-responsive element-binding proteins (DREBs) also play important roles in regulating water deficit stress responses in ABA-independent pathways (Umezawa et al., 2006). In this study, the *DREB* family member *NtDREB3* was induced in transgenic tobacco plants under drought stress, which indicated that *MdbHLH130* is involved in water stress through ABA-dependent and ABA-independent pathways (**Figure 7**). *NtLTP1* belongs to the lipid transfer protein family, which is universally involved in the response to ABA and drought stress (Hu et al., 2013; Wang et al., 2015). In the present study, *MdbHLH130-ox* transgenic tobacco plants showed enhanced *NtLTP1* expression under drought stress, suggesting that *MdbHLH130* affects the expression of some lipid-transfer protein genes that are responsive to water stress (**Figure 7**).

Water deficit stress often affects plant growth and development (Zhu, 2016). Apple trees are among the crops most affected by water stress, which significantly decreases fruit yield and quality (Reid and Kalcsits, 2020). It is well known that numerous genes are involved in drought tolerance. However, just few of them are characterized and used for genetic improvement in fruit tree. In summary, we isolated and characterized a dehydration stress-responsive bHLH TF (*MdbHLH130*) from apple, which acted as a positive regulator of water stress tolerance. We concluded that *MdbHLH130* exhibits important physiological functions in the water stress response through ABA signaling and antioxidant system regulation, thus preventing plants from oxidative damage. Our findings provide a deeper understanding of the regulatory mechanism of *MdbHLH130* in response to water deficit stress and may have candidate application for drought improvement in horticultural crop breeding. Further analyses of the direct downstream target genes of MdbHLH130 can help clarify its mechanisms in the enhanced tolerance to water stress.

## Data Availability Statement

The datasets generated for this study are available on request to the corresponding authors.

## Author Contributions

QZ and YW conceived and designed the research. QZ, ZF, LQ, QC, TW, and YL provided experimental materials. YW and QZ analyzed the data and wrote the manuscript. All authors contributed to the article and approved the submitted version.

## Funding

This work was supported by the National Key Research and Development Program of China (2018YFD1000300), Shandong Province Government (SDAIT-06-05), Breeding Plan of Shandong Provincial Qingchuang Research Team (2019), the Talents of High Level Scientific Research Foundation of Qingdao Agricultural University.

## Conflict of Interest

The authors declare that the research was conducted in the absence of any commercial or financial relationships that could be construed as a potential conflict of interest.
