# Supplementary material for: MdbHLH130, an Apple bHLH Transcription Factor, Confers Water Stress Resistance by Regulating Stomatal Closure and ROS Homeostasis in Transgenic Tobacco
Source: Front Plant Sci. 2020 Oct 9;11:543696. doi: 10.3389/fpls.2020.543696 (PMC7581937; doi:10.3389/fpls.2020.543696)
Supplement: Supplementary file 1 [file Table_1.docx]

***MdbHLH130*, an apple bHLH transcription factor, confers water stress resistance by regulating stomatal closure and ROS homeostasis in transgenic tobacco**

Qiang Zhao^1^*, Zihao Fan^1^, Lina Qiu^1^, Qinqin Che^1^, Yuanyuan Li^2^, Yongzhang Wang^1^*

^1^Shandong Collaborative Innovation Center of Fruit & Vegetable Quality and Efficient Production; College of Horticulture, Qingdao Agricultural University, Qingdao, Shandong 266109, China

^2^College of Horticulture Science and Engineering, Shandong Agricultural University, Tai-An, Shandong 271018, China

***Corresponding authors:**

1. Qiang Zhao

Address: College of Horticulture, Qingdao Agricultural University, Qingdao, Shandong 266109, China

E-mail address: [zhaoqiang000666@163.com](mailto:zhaoqiang000666@163.com)

2. Yongzhang Wang

Address: College of Horticulture, Qingdao Agricultural University, Qingdao, Shandong 266109, China

E-mail address: [qauwyz@163.com](mailto:qauwyz@163.com)


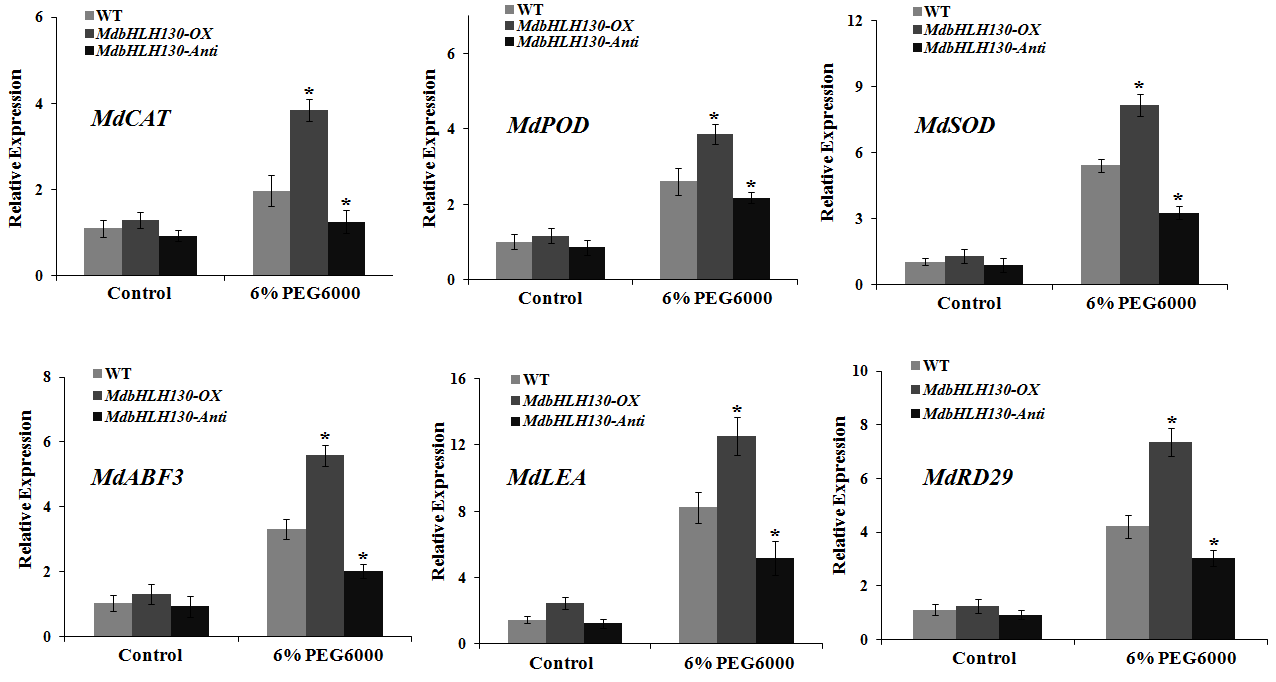


**Figure S1.** **The expression of the genes involved in ROS-scavenging and stress-responsive in the WT, *MdbHLH130-OX* and *MdbHLH130-Anti* transgenic apple calli.**

The expression levels were analyzed by qRT-PCR in WT, *MdbHLH130-OX* and *MdbHLH130-Anti* transgenic apple calli under control or PEG6000 treatment conditions. Error bars represent the means ±SD taken from three independent biological replicates. Asterisks indicate significant difference from WT (*P < 0.01).


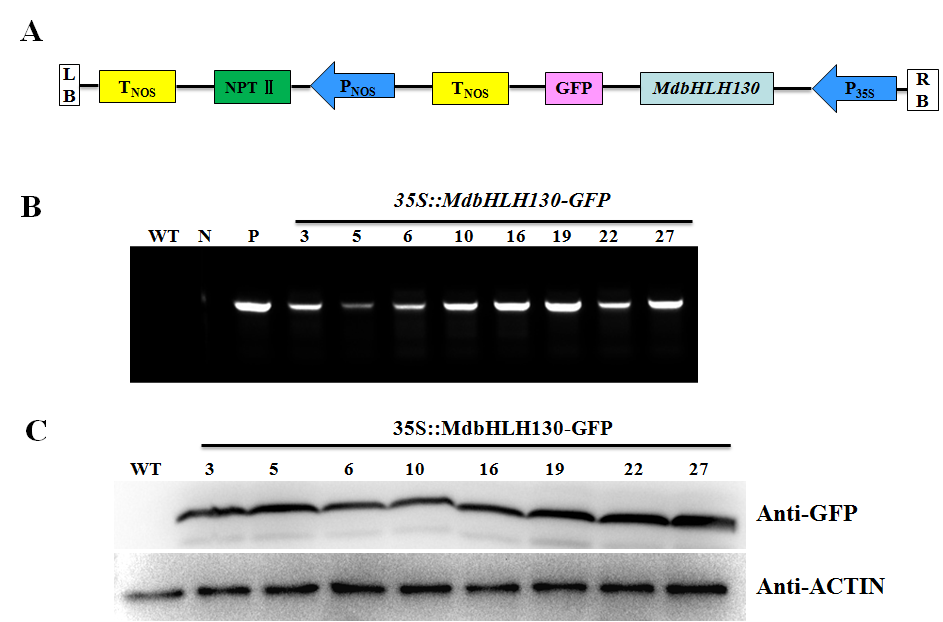


**Figure S2.** Identification of transgenic tobacco plants overexpressing *MdbHLH130*.

(A) Schematic diagram of the *35S::MdbHLH130-GFP* construct used for tobacco transformation.

(B) PCR confirmation of the *MdbHLH130-ox* plants. WT, wild type. N, used as a negative control. P, used as a positive control; the numbers indicate different transgenic lines.

C, Western blot analysis of MdbHLH130 in transgenic lines. Anti-ACTIN was used as an internal control.

**
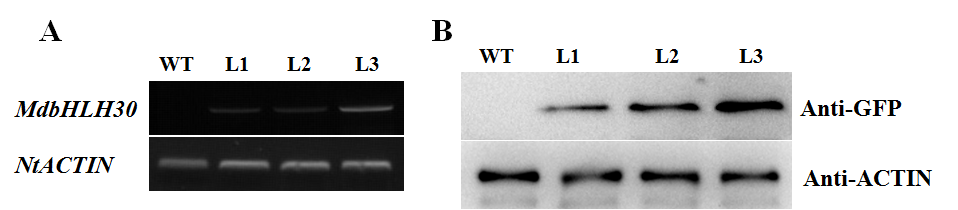
**

**Figure S3.** The expression levels of *MdbHLH130* in three transgenic tobacco lines.

(A) and (B) Relative expression and protein levels of MdbHLH130 in the WT and three transgenic tobacco lines (L1, L2 and L3) by semi-quantitative RT-PCR and western blot, respectively. *NtACTIN* and Anti-ACTIN was used as the internal control, respectively.

**
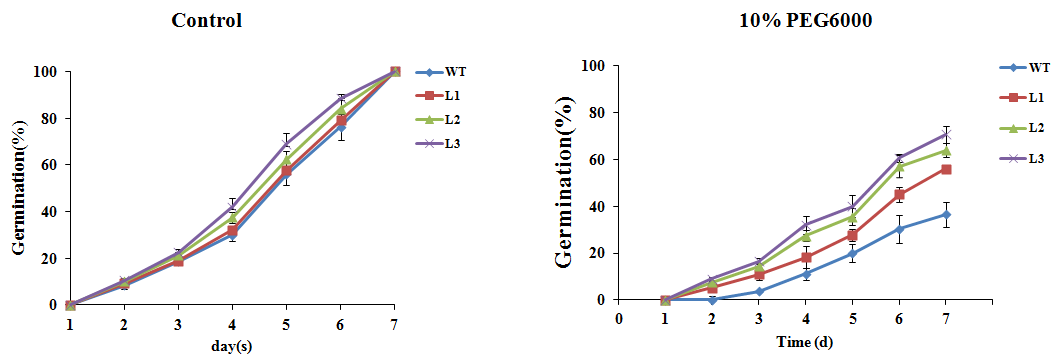
**

**Figure S4.** Analysis of germination in WT and transgenic lines exposed to 10% PEG6000 treatment.

Statistical analysis of the seed germination rate in WT and transgenic lines under control and 10% PEG6000 treatment conditions. Each value represents the mean ± SD of at least 100 seeds from three independent experiments.

**
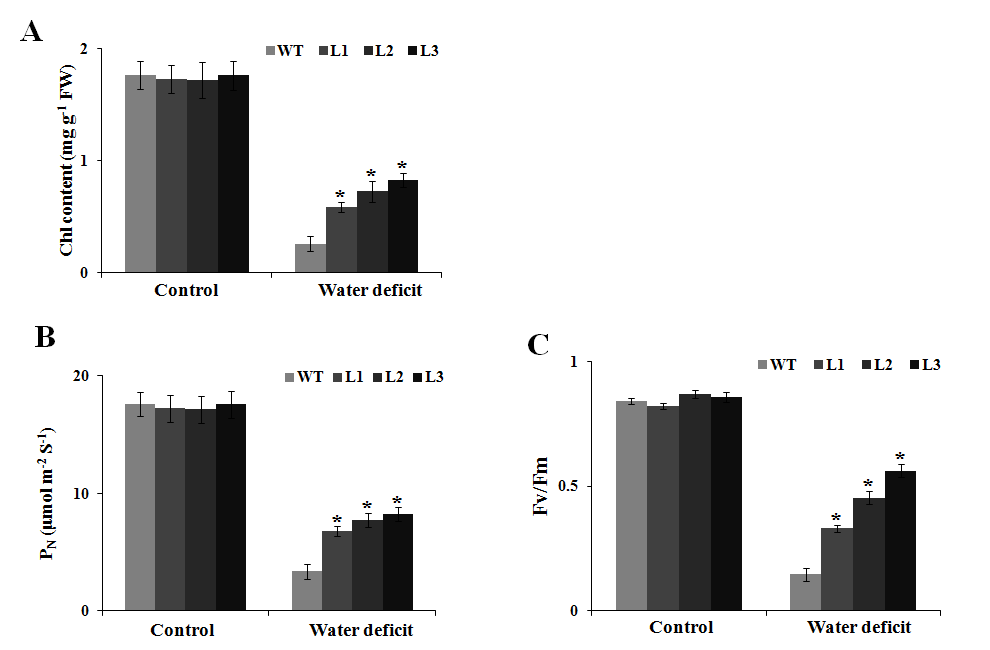
**

**Figure S5.** Effects of water deficit stress on the total chlorophyll content (A), P_N_ (B), and Fv/Fm (C) in both wild type (WT) and transgenic tobacco leaves. Data are the means of more than three replicates (± SD). Asterisks represent significant differences between WT and transgenic lines (*P < 0.01).

**Table S1.** Primers used for RT-PCR in this study.

| Gene |  | Primers |
| --- | --- | --- |
| *MdActin* |  | 5’-TGACCGAATGAGCAAGGAAATTACT-3’ |
|  |  | 5’-TACTCAGCTTTGGCAATCCACATC-3’ |
| *MdbHLH130* |  | 5’-TCTGGCTAGTGAAGGTGGTG-3’ |
|  |  | 5’-GACGACGGCTGGTAAAATCC-3’ |
| *NtActin* |  | 5’-CTATTCTCCGCTTTGGACTTGGCA-3’ |
|  |  | 5’-ACCTGCTGGAAGGTGCTGAGGGAA-3’ |
| *NtSOD* |  | 5’-CTCCTACCGTCGCCAAAT-3’ |
|  |  | 5’-GCCCAACCAAGAGAACCC-3’ |
| *NtPOD* | | 5’-GCTGTTCGACGAGTTGTTAACAG-3’ |
|  |  | 5’-CTCTGGCTGAGTTGTTGTTGG-3’ |
| *NtCAT* | | 5’-AGGTACCGCTCATTCACACC-3’ |
|  |  | 5’-AAGCAAGCTTTTGACCCAGA-3’ |
| *NtLEA5* | | 5’-TTGAATCTGGGGTTTTGGTT-3’ |
|  |  | 5’-GGAAGCATTGACGAGCTAGG-3’ |
| *NtERD10D* | | 5’-GAGGACACGGCTGTACCAGT-3’ |
|  |  | 5’-GCGCCACTTCCTCTGTCTT-3’ |
| *NtERD10C* | | 5’-AACGTGGAGGCTACAGATCG-3’ |
|  |  | 5’-GTTCCTCTTGGGCATGAGTT-3’ |
| *NtDREB3* | | 5’-GCCGGAATACACAGGAGAAG-3’ |
|  |  | 5’-CCAATTTGGGAACACTGAGG-3’ |
| *NtLTP1* | | 5’-GCAGAAGCCATAACCTGTGG-3’ |
|  |  | 5’-CAGTGGAAGGGCTGATCTTG-3’ |
| *NtNCED1* | | 5’-AAGAATGGCTCCGCAAGTTA-3’ |
|  |  | 5’-GCCTAGCAATTCCAGAGTGG-3’ |
| *MdCAT* | | 5’-CCGAACCCTAAGTCCCACATC-3’ |
|  |  | 5’- CTTGTGGAACTCCCAGGTCATC-3’ |
| *MdPOD* | | 5’- CCAACAAATGTGTCCCAAAAATG-3’ |
|  |  | 5’- CCTGGTCCGAGGTAAATAATCC-3’ |
| *MdSOD* | | 5’-TGAAGGGTGTTGCTGTTCTCG-3’ |
|  |  | 5’-ATGAAGTCCAGGCTTGAGGC-3’ |
| *MdABF3* | | 5’-CGAACGCTTAGTCAGAAA-3’ |
|  |  | 5’-AAAGTCCTCCAAAGTCATC-3’ |
| *MdLEA* | | 5’-TGGGGGAGATGACTTGGAG-3’ |
|  |  | 5’-CTGCTTCAGGTGTAGAAGC-3’ |
| *MdRD29* | | 5’-TGTGACAGGCGGTGAAGAAAT-3’ |
|  |  | 5’-TCAGCGATAGCGGAAGTGG-3’ |

**Table S2.** Primers used for plasmid construction in this study.

| Gene |  | Primers |
| --- | --- | --- |
| *MdbHLH130*  (Overexpression) |  | 5’-ATGGAATCAGATCTTCACCAG-3’ |
|  |  | 5’-CTGCTGCTTGTTTGAGCAAG-3’ |
| *MdbHLH130*  (Anti) |  | 5’-TTGCTCAAGGATTTTACCAG-3’ |
|  |  | 5’-AAATTTTTCAACCTGCTCGC-3’ |
| *P_MdbHLH130_::GUS* |  | 5’-CACGAATCCTATATATTCTTTC-3’ |
|  |  | 5’- CCTCTTCTATTTGGTTCAGCC-3’ |
| *MdbHLH130*  (pGBKT7) |  | 5’-ATGGAATCAGATCTTCACC-3’ |
|  |  | 5’-CTACTGCTGCTTGTTTGAGC-3’ |
